# Enhancement of Surfactin and Fengycin Production by* Bacillus mojavensis* A21: Application for Diesel Biodegradation

**DOI:** 10.1155/2017/5893123

**Published:** 2017-09-10

**Authors:** Noomen Hmidet, Hanen Ben Ayed, Philippe Jacques, Moncef Nasri

**Affiliations:** ^1^Laboratoire de Génie Enzymatique et de Microbiologie, Ecole Nationale d'Ingénieurs de Sfax, BP 1173, 3038 Sfax, Tunisia; ^2^ProBioGem-EA1026, Polytech'Lille, Université Lille-Nord de France, Villeneuve d'Ascq, France; ^3^TERRA Teaching and Research Centre, Microbial Processes and Interactions (MiPI), Gembloux Agro-Bio-Tech University of Liege, Gembloux, Belgium

## Abstract

This work concerns the study of the enhancement of surfactin and fengycin production by* B. mojavensis* A21 and application of the produced product in diesel biodegradation. The influences of the culture medium and cells immobilization were studied. The highest lipopeptides production was achieved after 72 hours of incubation in a culture medium containing 30 g/L glucose as carbon source and a combination of yeast extract (1 g/L) and glutamic acid (5 g/L) as nitrogen sources with initial pH 7.0 at 30°C and 90% volumetric aeration. The study of primary metabolites production showed mainly the production of acetoin, with a maximum production after 24 h of strain growth. The use of immobilized cells seemed to be a promising method for improving lipopeptides productivity. In fact, the synthesis of both lipopeptides, mainly fengycin, was greatly enhanced by the immobilization of A21 cells. An increase of diesel degradation capacity of approximately 20, 27, and 40% in the presence of 0.5, 1, and 2 g/L of produced lipopeptides, respectively, was observed. Considering these properties,* B. mojavensis* A21 strain producing a lipopeptide mixture, containing both surfactin and fengycin, may be considered as a potential candidate for future use in bioremediation and crop protection.

## 1. Introduction

Biosurfactants are produced by many species of yeast, bacteria, and filamentous fungi in the growth media or as part of the cell membrane [1]. They are amphiphilic compounds with hydrophilic and hydrophobic moieties. Biosurfactants have distinct advantages over their chemical equivalents with respect to their low toxicity, biodegradability, biocompatibility, ease of production from renewable resources, and high selectivity and stability at extreme temperature, pH, and salinity [2]. Among biosurfactants, lipopeptides are the most known and mainly produced by* Bacillus* [3],* Pseudomonas* [4],* Streptomyces* [5], and* Aspergillus* [6]. Structurally, they are composed of a lipid tail linked to a short linear or cyclic oligopeptide. Fengycin, surfactin, and iturin are the main lipopeptides families produced by* Bacillus* strains. They are made up of seven (surfactins and iturins) or 10 *α*-amino acids (fengycins) linked to one unique *β*-amino (iturins) or *β*-hydroxy (surfactins and fengycins) fatty acid. They are produced as mixtures of components varying in their peptidic and/or lipidic structure including homologous and isoform series, which differ in the length and branching of the fatty acid side chains and the amino acid substitutions in the peptide rings, respectively [7]. The application of lipopeptides in cosmetics product was highly increased due to their surface properties and diverse biological activities including antioxidant [8], antiwrinkle, and moisturizing activities on human skin [9]. In addition, they are used in environment and bioremediation applications due to their properties as viscosity reducers, hydrocarbon solubilizing, and metal sequestering candidates for application [10].

Numerous studies concerning free cells production of lipopeptides have been reported. Several parameters such as carbon sources, nitrogen sources, and temperature were studied [11, 12]. In addition, the production of biosurfactants by immobilized cells of* Bacillus subtilis* was well studied [13, 14].

The study of the structural analyses and biochemical characterization of lipopeptides produced by some* B. mojavensis* strains was also investigated. The crude lipopeptide mixture, containing surfactin and fengycin isoforms, of* Bacillus mojavensis* A21 strain was found to be very effective in reducing surface tension [15]. The endophytic bacterium* B. mojavensis* RRC1001 produced surfactin [16]. Ma et al. [17] reported that the strain* B. mojavensis* B0621A was found to produce a new iturinic lipopeptide called mojavensin A. However, few studies reported the influence of culture media on lipopeptides production by* Bacillus mojavensis* strains. In fact, maximum production of lipopeptides by* B. mojavensis* B0621A was performed at 28°C for 48 h under shaking at 180 rpm in sucrose mineral salt medium [18].

In this study, the effect of medium components, environmental conditions, and cells immobilization on surfactin and fengycin coproduction by* B. mojavensis* A21 was studied. The ability of A21 lipopeptides to enhance diesel biodegradation by A21 strain was also reported.

## 2. Materials and Methods

### 2.1. Bacterial Strain

*B. mojavensis* (EU366229) strain A21 used in the present work was isolated in our laboratory from marine water in Sfax City [19].

### 2.2. Medium Composition and Culture Condition

The studied strain was grown in Luria-Bertani (LB) broth medium [20]. The medium used for surfactin and fengycin coproduction was composed of (g/L) carbon source (glucose, fructose, sucrose, galactose, or starch), nitrogen source (yeast extract, soya peptone, glutamic acid, aspartic acid, ammonium sulphate, or ammonium nitrate), and salts (K_2_HPO_4_, 1 g/L; MgSO_4_·7H_2_O, 0.5 g/L; KCl, 0.5 g/L; CuSO_4_, 1.6 mg/L; Fe_2_(SO_4_)_3_, 1.2 mg/L; and MnSO_4_, 0.4 mg/L). The strain was cultured in 25 ml of medium in 250 ml conical flasks maintained at 30°C and 160 rpm for 72 h.

### 2.3. Optimization of Surfactin and Fengycin Coproduction

The effects of different carbon sources (glucose, fructose, sucrose, galactose, and starch) on surfactin and fengycin coproduction by* B. mojavensis* A21 were examined at a concentration of 2%. The nitrogen source used was yeast extract, 1 g/L, and glutamic acid, 5 g/L. The best carbon source was further optimized in the range of 1 to 6%.

To investigate the effect of nitrogen source on surfactin and fengycin coproduction, the combination of glutamic acid and yeast extract in the basal medium, containing 20 g/L glucose, was replaced with different organic (yeast extract, soya peptone, glutamic acid, and aspartic acid) and inorganic (ammonium sulphate and ammonium nitrate) compounds at a concentration of 5 g/L. Other combinations (yeast extract, 1 g/L, + glutamic acid, 5 g/L, yeast extract, 1 g/L, + aspartic acid, 5 g/L, and yeast extract, 1 g/L, + glutamic acid, 5 g/L, + ammonium sulphate, 1 g/L) were also tested.

The effect of incubation temperature on surfactin and fengycin coproduction was also investigated at different temperatures (20, 30, and 37°C).

To investigate the effect of pH on lipopeptides production, the initial pH of the medium was adjusted from 5.0 to 9.0. The effect of aeration was also studied by varying the volume of headspace in 250 ml flasks and expressed as volumetric percentage of medium per flask volume (10, 20, and 30%).

### 2.4. HPLC Analysis of Glucose Organic Acids and Acetoin

Glucose, lactate, acetate, and acetoin concentrations were analyzed in the filtered samples by HPLC using a Fast Fruit Juice column. The elution was performed using phosphoric acid (0.05%) with a flow rate of 0.8 ml/min at 55°C.

### 2.5. Effect of Cells Immobilization on Surfactin and Fengycin Coproduction

The coproduction of surfactin and fengycin was performed with polypropylene solid carriers coated with Fe^2+^ support. Production of lipopeptides using immobilized cells occurs in two steps: colonisation experiment and production experiment.

#### 2.5.1. Colonisation of Support

The effect of inoculum size (OD = 0.1, 0.3, and 0.5) and period of the contact with support (48, 96, and 144 h) on biomass yield (cells/g of beads) was studied. The capacity of colonisation was studied using 40 g of beads in 25 ml of modified Landy medium (pH 7 buffered with MOPS 100 mM without glutamic acid) in 250 ml conical flasks at 30°C and agitation at 40 rpm.

For biomass quantification after cells colonisation, beads were removed and sonicated in 4 ml of water during 2 min. Then biomass was estimated as viable cells by plate count method.

#### 2.5.2. Production of Lipopeptides

The cultures were inoculated at OD = 0.5 as initial optical density (600 nm) and after 144 h of colonisation on beads. The beads were gently washed in phosphate buffer and replaced in lipopeptides production medium in 25 ml of medium in 250 ml conical flasks. The culture was agitated at 160 rpm and 30°C during 72 h.

### 2.6. Quantitative Analysis of Surfactin and Fengycin

High-performance liquid chromatography (C18 column) technique was used for the measurement of surfactin and fengycin concentrations. The surfactin was eluted under acetonitrile (80%), water (20%), and trifluoroacetic acid (0.1%) at 0.6 ml/min. Fengycins were eluted under a gradient acetonitrile/H_2_O/trifluoroacetic acid from 45/55/0.1 to 55/45/0.1 at 0.6 ml/min. Spectra were analyzed using values of second derivative with a major peak at 212-213 nm associated with a minor peak for surfactin [21] and two major peaks at 213 and 236 nm associated with a minor peak at 290 nm for fengycin [22].

### 2.7. Surfactin and Fengycin Extraction

The lipopeptides produced by* B. mojavensis* A21 strain were prepared using ultrafiltration method (10-kDa membrane) and solubilization of the retentate by methanol. Purified lipopeptides were obtained after a second ultrafiltration step and evaporation.

### 2.8. Effect of A21 Lipopeptides on Diesel Biodegradation

The effect of A21 lipopeptides on diesel biodegradation was tested using three concentrations (0.5, 1, and 2 mg/mL). Cultures were performed in Erlenmeyer flasks containing 25 mL of mineral salts medium composed of (g/L) (NH_4_)_2_SO_4_ 1, CaCl_2_ 0.01, KH_2_PO_4_ 0.5, MgSO_4_ 7H_2_O 0.25, and KCl 0.25 and 2 mL trace element stock solution containing (mg/L) FeSO_4_ 15.2, MnSO_4_ H_2_O 1.51, CuSO_4_ 5H_2_O 0.16, and ZnSO_4_ 7H_2_O 0.16, supplemented with crude oil (diesel) at a final concentration of 3.0% (v/v). Uninoculated flask served as control. Cultures were incubated at 30°C and 160 rpm for 15 days.

Then whole cultivation broth was centrifuged and the residual crude oil was recovered by hexane (25 mL) extraction. The solvent was allowed to evaporate and the total biodegradation percentage was determined gravimetrically using the expression described by Chaillan et al. [23].

(1) $$\begin{array}{c} \text{Biodegradation percentage}=\left(\;\frac{\left(W_{c}-W_{1}\right)}{W_{c}}\;\right)\times 100, \end{array}$$

where *W*_1_ is the weight of the residual oil in sterile control and *W*_*c*_ is the weight of the fraction in the culture.

## 3. Results and Discussion

### 3.1. Surfactin and Fengycin Coproduction by* B. mojavensis* A21

Previously Ben Ayed et al. [15] reported the detection and identification of pumilacidin, surfactin, and fengycin isoforms produced by* B. mojavensis* A21 strain. We first checked the lipopeptide synthesis potential by polymerase chain reaction (PCR) using a set of specific degenerated primers. MALDI-TOF mass spectrometry has been used as a second method for identification and structural characterization of produced lipopeptides.

**Figure 1:**
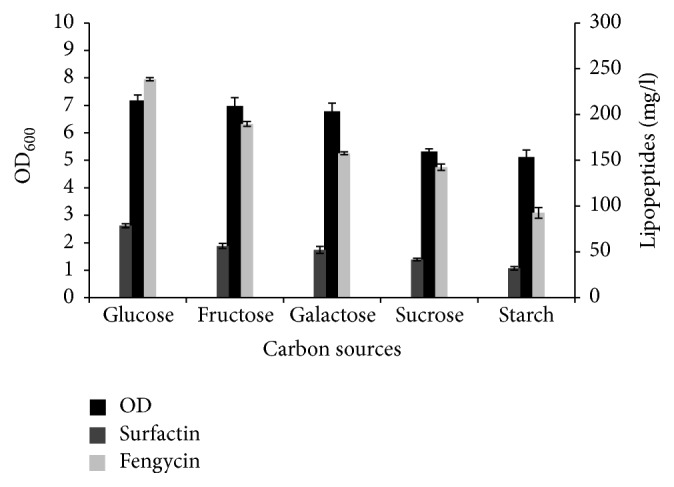
Effect of different carbon sources on growth and lipopeptides production by* B. mojavensis* A21. The strain was cultured in 25 ml of medium in 250 ml conical flasks maintained at 30°C and 160 rpm for 72 h. The nitrogen source used was yeast extract, 1 g/L, and glutamic acid, 5 g/L.

Many studies have pointed out different environmental factors for their effect on lipopeptides production [24, 25]. Because carbon source is considered as important parameter for lipopeptides production,* B. mojavensis* A21 strain was grown in the presence of different carbon source at a concentration of 20 g/l. The microbial growth kinetics and lipopeptides production are represented in Figure 1. The production of both surfactin and fengycin was observed in all used carbon sources. However, the best production was obtained with glucose. However, lipopeptides production was significantly low when the strain was grown on starch.

These results corroborate well with previous studies showing that glucose was the most preferred substrate for lipopeptides production. Addition of glucose resulted in a higher specific production rate of lipopeptides (lipopeptides/cells) by* B. subtilis* C9, more than the use of sucrose [26]. Glucose has also been mentioned as efficient carbon source for lipopeptides synthesis by* B. subtilis* SPB1 [11]. In contrast to our results, sucrose was found to be the best carbon source for fengycin production by* B. subtilis* S499 [25].

**Figure 2:**
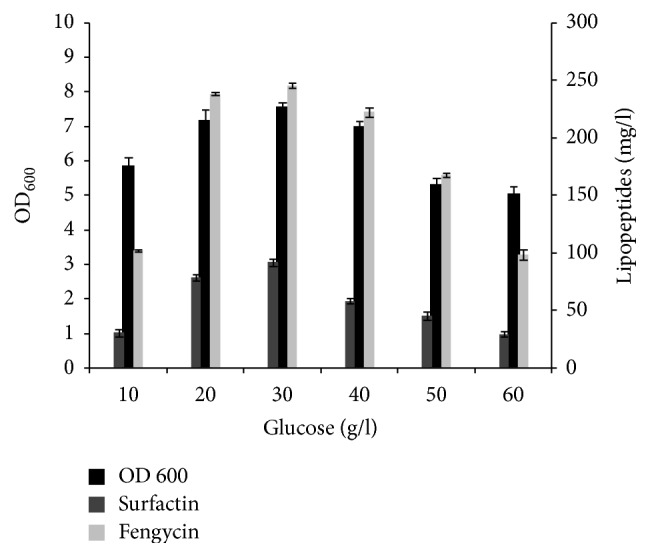
Effect of different concentrations of glucose, on growth and lipopeptides production by* B. mojavensis* A21. Glucose concentration was tested in the range of 1 to 6%.

Since the production medium was optimized with glucose as sole carbon source, different concentrations of glucose were examined for the best yield of lipopeptides from the studied strain (Figure 2). The obtained results showed an increase in lipopeptides concentration with increasing the initial glucose concentration up to 30 g/L. However, further increase of glucose concentration resulted in decrease of lipopeptides production. Similar results were found by Ghribi and Ellouze-Chaabouni [11], showing that biosurfactants concentration was increased with increasing the initial glucose concentration with a maximum at 40 g/L and then was decreased slightly (96%). This result could be explained by the fact that the produced cells at high glucose concentrations are not physiologically adequate for synthesizing biosurfactants [11].

**Figure 3:**
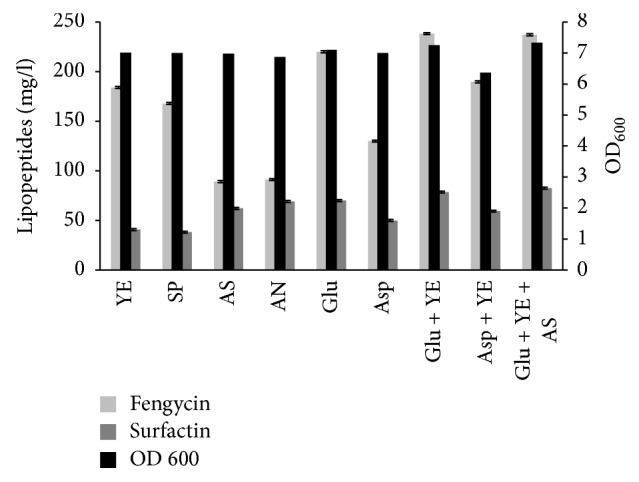
Effect of different nitrogen sources on growth and lipopeptides production by* B. mojavensis* A21. YE: yeast extract, SP: soya peptone, AS: ammonium sulphate, AN: ammonium nitrate, Glu: glutamic acid, and Asp: aspartic acid. The combination of glutamic acid and yeast extract in the basal medium, containing 20 g/L glucose, was replaced with different nitrogen source at a concentration of 5 g/L. Other combinations (yeast extract, 1 g/L, + glutamic acid 5 g/L, yeast extract, 1 g/L, + aspartic acid, 5 g/L, and yeast extract, 1 g/L, + glutamic acid, 5 g/L, + ammonium sulphate, 1 g/L) were also tested.

Another optimizing agent which was utilized for lipopeptides production was nitrogen source that plays role in cellular metabolism thus affecting its production. In the present study, various nitrogen sources were tested. The results obtained (Figure 3) showed that the highest lipopeptides production determined in the culture broth was obtained when a combination of glutamic acid (5 g/L) and yeast extract (1 g/L) was used as nitrogen sources. Moreover, compared to the organic nitrogen sources which support good growth and lipopeptides production, inorganic nitrogen sources like ammonium sulphate and ammonium nitrate are not efficient for lipopeptides production. The results revealed also that no enhancement of lipopeptides production was gained by the replacement of glutamic acid by aspartic acid. These results are in agreement with those previously obtained by Chtioui et al. [13] showing that the Landy medium which contains glutamic acid as nitrogen source promotes the synthesis of lipopeptides by* B. subtilis.* Other nitrogen sources have been also reported to support lipopeptides production by some* Bacillus* strains like urea [27] and ammonium nitrate [28].

The effects of temperature, initial pH, and aeration on surfactin and fengycin coproduction by* B. mojavensis* A21 were studied in optimized medium containing glucose (30 g/L), glutamic acid (5 g/L), and yeast extract (1 g/L).

**Figure 4:**
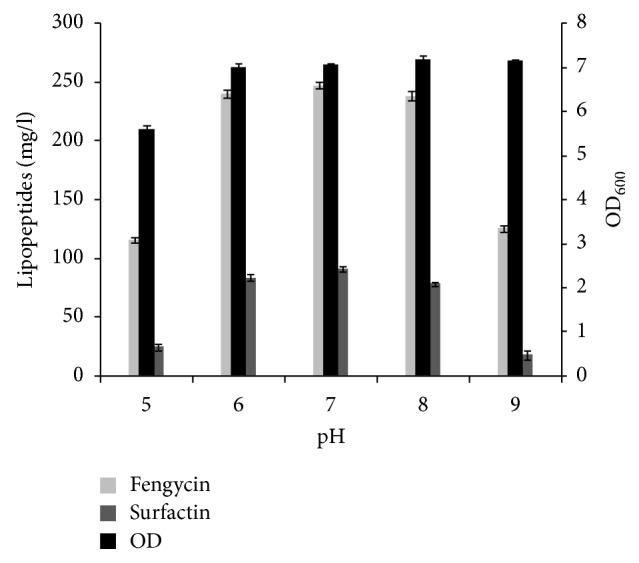
Effect of different initial pH values of fermentation medium on growth and lipopeptides production by* B. mojavensis* A21. The strain was cultured in 25 ml of medium in 250 ml conical flasks maintained at 30°C and 160 rpm for 72 h. The nitrogen source used was yeast extract, 1 g/L, and glutamic acid, 5 g/L, and glucose, 20 g/L.

The pH of medium has been shown to affect many enzymatic processes and transport of compounds across the cell membrane and therefore the production process. In this study, the effect of pH on lipopeptides production was investigated at different pH values (5.0–9.0). As shown in Figure 4, the production levels of surfactins and fengycins were increased by a factor of 3.7 and 2.13, respectively, when increasing the pH value from 5.0 to 7.0. Earlier studies revealed that neutral initial pH favoured lipopeptides production by* Bacillus* strains [14, 29, 30].

To study the effect of temperature on lipopeptides production, the strain was grown at different temperatures (20, 30, and 37°C) for 72 h. The results revealed that optimum level of surfactin (127.14 mg/L) was achieved at 20°C. However, the best fengycin production was obtained at 30°C (Table 1). However, higher temperature (37°C) unfavoured lipopeptides synthesis by A21 strain. Numerous studies have pointed out the effect of temperature on lipopeptides production. For example, higher temperature (37°C) favoured surfactin synthesis of* B. subtilis* RB14 isolated from compost [31] but not that of* Bacillus subtilis* S499 [25]. In other hand, a 30-fold increase in mycosubtilin production was observed when the temperature decreased from 37 to 25°C; this was observed for both strain* B. subtilis* ATCC6633 and its derivative BBG100, which is a constitutive overproducer [32].

**Figure 5:**
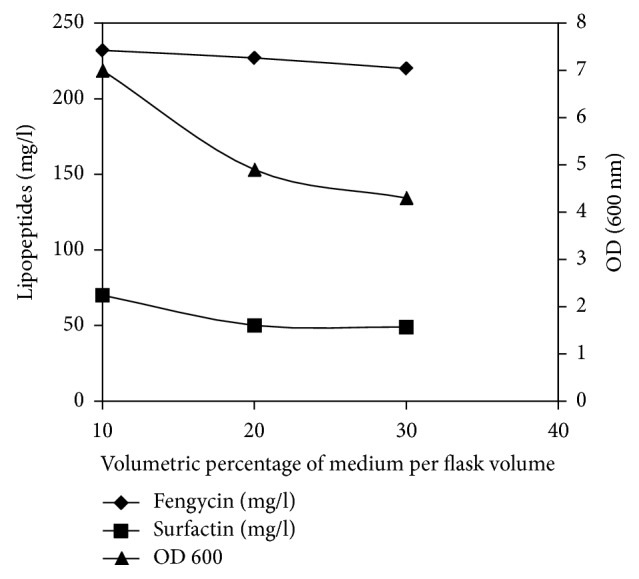
Effect of aeration on growth and lipopeptides production by* B. mojavensis* A21.

The effect of aeration on lipopeptides production was tested in 250 ml flasks at different relative filling volume. The highest production of surfactin and fengycin was observed in 250 ml flasks with 25 ml of culture medium. A sharp decline in this production occurred upon decreasing of aeration upon 70% (250 ml flasks with 75 ml of culture medium) (Figure 5). In contrast, there are no significant differences between fengycin production observed in the three different relative filling volumes. These results are in agreement with those previously obtained by Jacques et al. [25] showing that* B. subtilis* S499 produce more surfactin in better aeration condition. In the same context, Chollet-Imbert et al. [22] showed that* B. subtilis* ATCC9943 produced more fengycin when oxygen was limited.

### 3.2. HPLC Analysis of Glucose and Primary Metabolites Production

**Figure 6:**
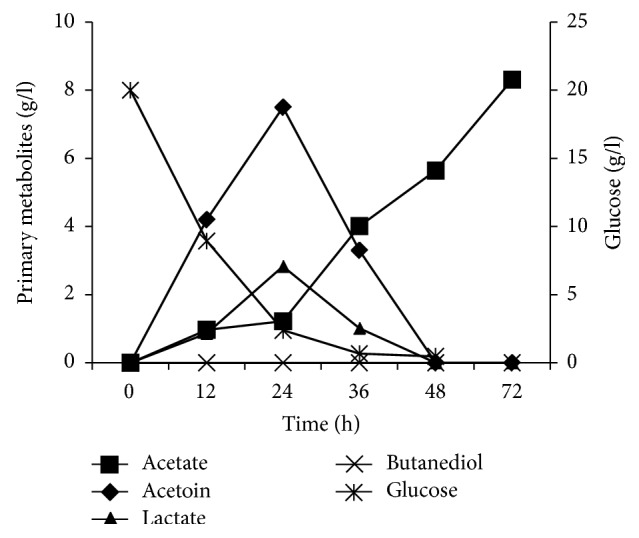
Glucose consumption and primary metabolites production by* B. mojavensis* A21.

*Bacillus* strains are known to produce acetoin and 2,3-butanediol, which have been proved to elicit induced systemic resistance (ISR) in plants [33]. In this study, glucose consumption and the consequent production of metabolites like lactate, acetate, acetoin, and butanediol were studied (Figure 6).

Lactate production reached 2.83 g/L after 24 h of culture. In addition, acetoin production reached its maximum level after 24 h of growth (7.5 g/L) and then showed a slight decrease and completely converted after 48 h. Similar patterns were observed for the production of these metabolites by* Bacillus amyloliquefaciens* OG [34]. The primary metabolite production has been demonstrated previously with other strains of* Bacillus* using glucose as the main source of carbon and limited oxygen [35, 36]. Acetoin production is stimulated by pyruvate when glucose is present as an energy source and contributes to maintaining internal pH of cell [37]. Analysis of organic acids production shows also a slight lactate production that reaches its highest point at the end of exponential phase and is then used after glucose has been depleted. It has been proposed that a large percentage of glucose added to the culture was converted to acetate during vegetative growth [38].

However, the lack of 2,3-butanediol seems as an unexpected result. It has been shown that the acetoin utilization occurs after acetate depletion and then its reduction generated 2,3-butanediol which plays an important role in the cell.

The coproduction of lipopeptides and acetoin improved the use of A21 extract which can be used in crop protection. In addition, acetoin (3-hydroxybutan-2-one) is widely used in food, cigarettes, flavour, cosmetics, detergents, and chemical synthesis [39]. The absence of 2,3-butanediol facilitates the recovery of acetoin, which can be easily performed using an acetone/phosphate aqueous two-phase system [40].

### 3.3. Surfactin and Fengycin Coproduction by Immobilized Cells of* B. mojavensis* A21

Immobilization of cells was proved to be an efficient method to improve lipopeptides production [13, 14]. Lipopeptides production by immobilized cells occurs in two steps: colonisation step and production step.

**Figure 7:**
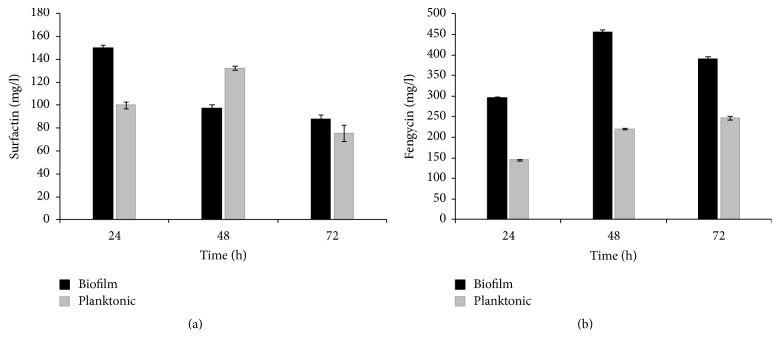
Production of lipopeptides by* B. mojavensis* A21 (optimized medium, 30°C, 144 h of colonisation; polypropylene solid carriers coated with Fe^2+^ support were used as support): (a) surfactin production; (b) fengycin production.

The maximal biomass (10^8^/g of support) was obtained after 144 h of contact with support and with initial OD of 0.5 (Table 2). The production of lipopeptides was performed using support under these conditions (Figure 7). In fact, the beads were replaced in lipopeptides production medium and incubated at 30°C during 72 h. As reported in Figure 7, the immobilized cells improve highly the fengycin and surfactin coproduction, especially at the beginning of fermentation process, while the concentrations are relatively low. In fact, after 24 h, biofilm cultures produced more lipopeptides than planktonic cells. This effect still exists at 48 h and 72 h in the case of fengycin. The effect of addition of polypropylene solid carriers coated with Fe^2+^ was more important on fengycin concentrations; the fengycin production increases up to 2 times after the immobilization. These results are in agreement with those previously obtained by Gancel et al. [14] showing that immobilized cells of* B. subtilis* BBG21 produce more lipopeptides than planktonic cells.

However, the production of both surfactin and fengycin was decreased after 72 h of cultivation. This result was similar to result found by Leclère et al. [41]. In fact, the biofilm surface becomes more hydrophobic, which causes lipopeptide production decrease.

### 3.4. Application of A21 Lipopeptides in Diesel Biodegradation

Surface-active compounds, like lipopeptides, can improve the solubility and removal of hydrophobic soil contaminants and, consequently, their biodegradation. In this study A21 strain was tested for its ability to degrade and utilize diesel fuel as the sole carbon and energy source in the presence of different concentrations of lipopeptides (0.5, 1, and 2 g/L). Control medium without lipopeptides addition was used. Cultures were performed in diesel oil (3%) based medium at 30°C for 15 days. Different samples were taken at regular intervals and diesel degradation percentage and viable cells were determined.

**Figure 8:**
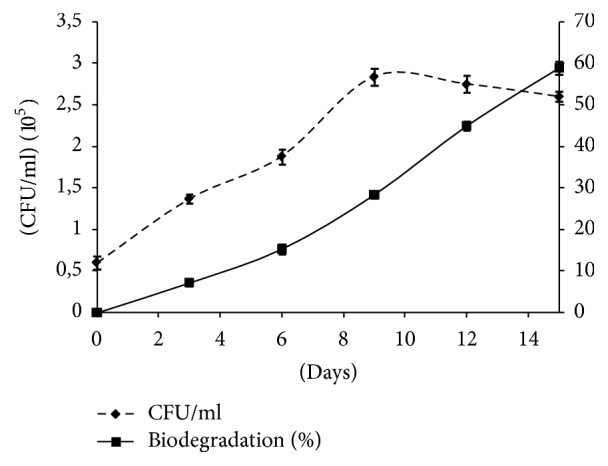
Kinetics of strain growth and diesel oil biodegradation. Strain growth was performed at 30°C and 160 rpm.

As reported in Figure 8, the strain was able to grow in the presence of diesel and without A21 lipopeptides (control medium). The biomass of* B. mojavensis* A21 increased exponentially with the increase in incubation time up to 8 days and declined thereafter.

The biodegradation percentage reached 15% after six days and thereafter increased till a maximum value (about 58%) after 15 days of incubation.

The addition of A21 lipopeptides at different concentrations to the diesel based medium showed an increase of diesel degradation capacity with the increase of lipopeptides concentration. In fact, compared with the lipopeptides-free inoculated culture, a significant increase of the biodegradation efficiency of diesel oil by the A21 strain, of approximately 20, 27, and 40% in the presence of 0.5, 1, and 2 g/L of lipopeptides, respectively, was observed.

The improvement of diesel biodegradation by lipopeptides addition was described previously [42, 43]. The mechanism proposed for improving degradability of hydrophobic organic compounds includes the ability of biosurfactants to enhance the bioavailability of the hydrocarbon through pseudosolubilization, which effectively reduces the interfacial tension between this compound and bacterial cell surfaces and probably facilitates the take-up of this substrate by the organism.

## 4. Conclusion

The nature of culture conditions and composition of media for optimal production of lipopeptides by* B. mojavensis* A21 have been developed in this study. Glucose and glutamic acid showed significant effect on lipopeptides production. The optimum initial pH of medium and incubation temperature for maximum production were found to be 7.0 and 30°C. The immobilization of* B. mojavensis* A21 on supports of low density allows a higher production of both surfactin and fengycin and could improve mainly fengycin production.

The study of primary metabolites production showed interestingly the production of acetoin, a well-known molecule implicated in plant defense. The addition of lipopeptides at different concentrations to the diesel based medium increased diesel degradation by A21 strain.

In conclusion, surfactin and fengycin were coproduced with acetoin, which gives more added value to the produced extract of* B. mojavensis* A21.

## Figures and Tables

**Table 1:** Effect of temperature on surfactin and fengycin coproduction by *B. mojavensis* A21.

Temperature (°C)	20	30	37
OD_600_	6.68	7.07	7.57
Surfactin (mg/L)	127.14	91.96	42.7
Fengycin (mg/L)	186.1	247.3	96.14

The strain was cultured in 25 ml of medium in 250 ml conical flasks maintained at different temperatures (20, 30, and 37°C) and 160 rpm for 72 h.

**Table 2:** Quantification of biomass immobilized on supports.

Inoculation	OD = 0.1	OD = 0.3	OD = 0.5
48 h	15,10^6^	29,10^6^	48,10^6^
96 h	38,10^6^	70,10^6^	95,10^6^
144 h	44,10^6^	78,10^6^	100,10^6^

Polypropylene solid carriers coated with Fe^2+^ support in Landy base medium at pH 7 buffered with MOPS 100 mM without glutamic acid.
